# Delayed Self‐Pollination as the Primary Reproductive Strategy in *Cypripedium shanxiense* S. C. Chen: Observations From Beijing Songshan National Nature Reserve

**DOI:** 10.1002/pei3.70069

**Published:** 2025-07-23

**Authors:** Qun Wang, Jing An, Meihong Zhu, Xing Zhang, Nan Wang, Baoqiang Zheng

**Affiliations:** ^1^ Key Laboratory of Tree Breeding and Cultivation of National Forestry and Grassland Administration Research Institute of Forestry, Chinese Academy of Forestry Beijing China; ^2^ Beijing Songshan Natural Reserve Administration Beijing China; ^3^ Xinjiang Forestry Vocational School Xinjiang China

**Keywords:** *Cypripedium shanxiens*e S. C. Chen, pollinator, seed setting rate, self‐pollination

## Abstract

Studying the pollination biological characteristics of the rare and endangered plant *Cypripedium shanxiense* S. C. Chen will provide a scientific basis for formulating conservation strategies for the species. This study investigates the flowering phenology, pollination insects, and the process of pollination and fruiting of *C. shanxiense* at the Beijing Songshan National Nature Reserve. The results indicate that the flowering period of *C. shanxiense* is 12–16 days. The full bloom period extends from early June to late June, with the population flowering duration lasting about 24 days. Four days after flowering, the pollen changes from a mucilaginous to a liquid state and flows to the stigma under the force of gravity. *C. shanxiense* exhibits high self‐compatibility and can produce seeds through self‐pollination, with a natural seed setting rate of 70%. *Lasioglossum zonulum* was observed visiting flowers and capable of carrying away pollen; its size traits related to pollination function are well matched with the flower structure of *C. shanxiense*, but its visiting frequency is extremely low. Overall, delayed self‐pollination is the primary pollination method for *C. shanxiense*, rather than insect‐assisted pollination.

## 1 Introduction

Orchids, with their unique charm and highly evolved characteristics, have long attracted the attention of scientists in botanical research (Wang et al. 2018, 2024; Yang et al. 2021). *Cypripedium shanxiense* S. C. Chen, a precious species of the Orchidaceae *Cypripedium*, has slipper‐shaped flowers with bright colors, possessing high ornamental value PEVuZE5vdGU (Chen et al. 2009; Wu et al. 2023). However, due to habitat destruction, over‐collection, and other reasons, the wild resources of *C. shanxiense* are increasingly diminishing, and its survival status is concerning (Wang et al. 2025). Currently, research on *C. shanxiense* is relatively scarce. Understanding the reproductive biological characteristics of *C. shanxiense* is of great significance for formulating effective conservation strategies.

In the reproductive process of plants, pollination is one of the most crucial steps. The plant pollination process involves the interactions between plants and pollinators, the adaptive changes in floral morphological structures, and the influence of environmental factors. Significant achievements have been made in the study of the pollination biology of some *Cypripedium* plants, such as *C. guttatum* (Bänziger et al. 2005), *C. tibeticum* (Li et al. 2006), *C. yunnanense* (Banziger et al. 2008), *C. subtropicum* (Jiang et al. 2021), and *C. lichiangense* (Zheng et al. 2022). Most of them attract pollinators using deceptive methods, such as scent, color, and traps. However, the pollination biology of *C. shanxiense* remains a blank area of research.

In addition to insect‐mediated cross‐pollination, self‐pollination, as a special form of pollination, also plays a significant role in the reproductive strategies of orchids (Suetsugu 2015; Van der Niet 2018). Self‐pollination can be divided into two types: immediate self‐pollination and delayed self‐pollination. Immediate self‐pollination refers to the process where flowers self‐pollinate immediately after opening, while delayed self‐pollination occurs when self‐pollination happens after the flower has been open for some time due to external environmental factors or physiological changes within the plant (Zhou et al. 2012; Suetsugu 2013). The mechanism of self‐pollination can increase the reproductive success rate of orchids in environments where pollinators are scarce (Suetsugu 2015). Currently, the phenomenon of self‐pollination has been observed in many orchid species and has attracted widespread attention from researchers.

The floral characteristics of *C. shanxiense* exhibit distinct adaptability, such as the unique shape, color, and patterns of the labellum, which undoubtedly play a significant role in attracting potential pollinators. However, the natural environment is not always favorable and unchanging. Fluctuations in climate, dynamics of pollinator communities, and disturbances from other biological or abiotic factors can all impact the pollination process of *C. shanxiense*. Therefore, we conducted in‐depth observations and analyses of the pollination biology of *C. shanxiense* in the Beijing Songshan National Nature Reserve, aiming to explore the pollination mechanism of this species and provide a theoretical basis for its conservation and breeding.

## 2 Materials and Methods

### 2.1 Study Sites

**FIGURE 1:**
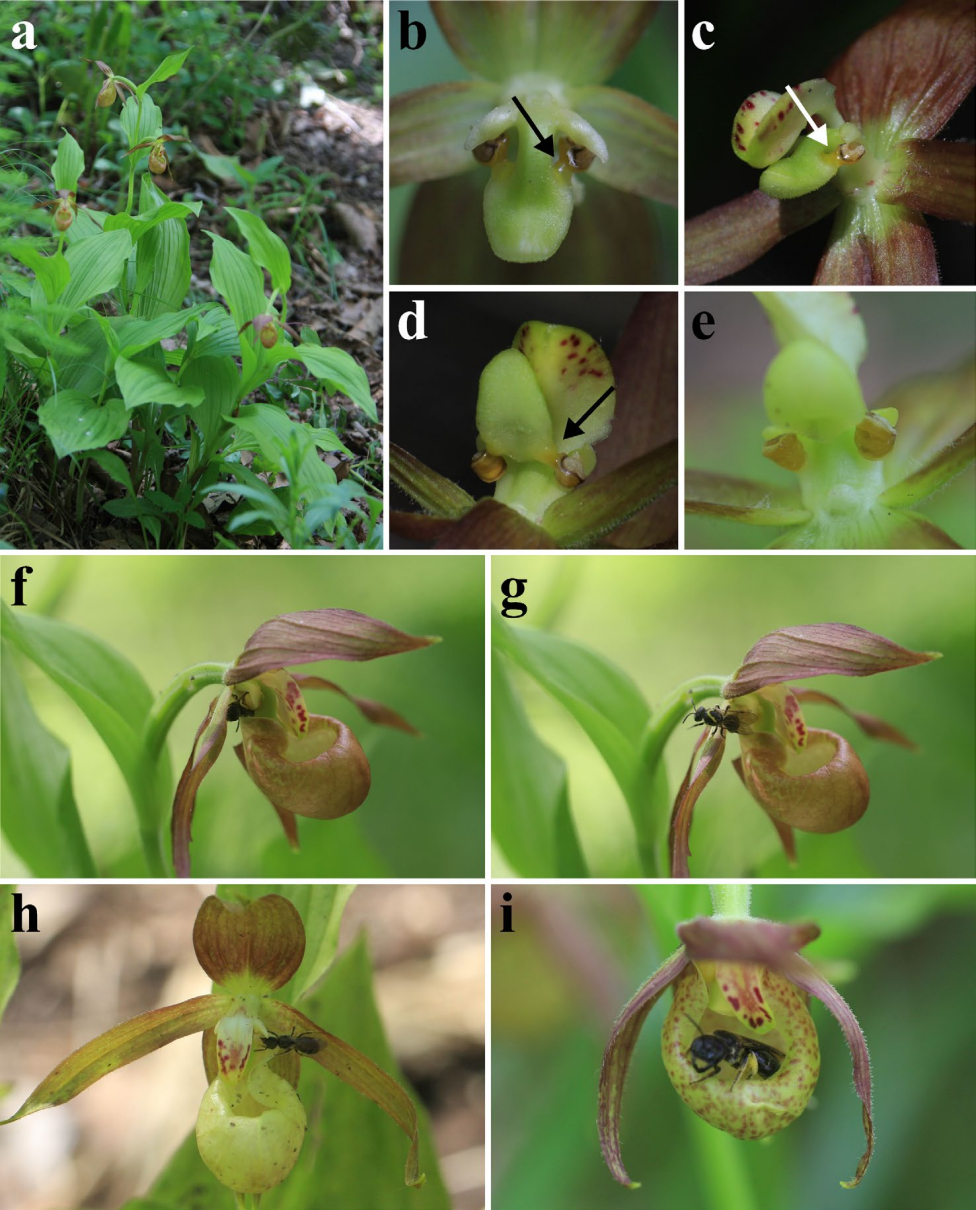
*C. shanxiense* and its pollinator *L. zonulum* . (a) *C. shanxiense* and its habitats. (b–d) Pollen became liquid and flowed to the stigma (these images were vertical view, horizontal view, and upward view after labellum removal respectively. The arrow pointed to pollen). (e) If the stigma was higher than the pollen mass, liquid pollen could not flow to the stigma and self‐pollination could not be completed as well (viewed upward after removal of labellum). (f) *L. zonulum* extruded from the exit of the pollination channel of *C. shanxiense*; (g) *L. zonulum* carried pollen mass (as shown by arrow). (h) *L. zonulum* could also contact the pollen mass from the exit of the pollination channel. (i) *L. zonulum* exited from the entrance of the pollination channel.

The field observation and experiment site was located in Yudu Mountain of Songshan International Nature Reserve in Beijing. The reserve is in the northwest of Beijing, with an altitude ranging from 627.6 to 2241 m, an annual average temperature of 8.86°C, and an annual average rainfall of 452.5 mm. The populations of *C. shanxiense* largely grow on the margin of the birch grove ( *Betula platyphylla* ) (Figure 1a).

### 2.2 Observation of Flowering Phenology

Fifteen plants exhibiting consistent growth conditions within the study area were selected through random sampling and marked. Cameras were set up to observe their flowering phenology, which included recording the flowering date, individual flower duration, and population flowering duration. Full bloom was determined when the petals were fully opened, visible, and accessible to pollinating insects. Flowers were excluded from observations once their petals had faded and withered, at which point they lost their attractiveness to pollinators or were no longer easily accessible to them.

### 2.3 Flower Visiting Behavior of Pollinators

At the full‐bloom stage, the pollination of *C. shanxiense* was observed from 8:30 a.m. to 17:00 p.m. on sunny days for a total of 8 days. Many behaviors of visiting insects were registered in detail and recorded with photos. The behaviors included the behavior before and during the process of visiting flowers, the escape, and other behaviors, as well as the visiting duration for each flower. Insects were classified as pollinators if they carried *C. shanxiense* pollen on their bodies. Visiting insects were captured for identification by entomologists at the Research Institute of Forest Ecology, Environment and Protection, Chinese Academy of Forestry. The insect specimens were stored in the herbarium of the Research Institute of Forestry, Chinese Academy of Forestry, and can be accessed through the contact person Baoqiang Zheng (zhengbaoqiang@aliyun.com) under the registration number LZ0001.

### 2.4 Morphological Matching Between Flowers and Pollinators

In order to evaluate the morphological matching relationship between flowers and their pollinators, a sample of five flowers at peak bloom for 3 days was randomly selected. The main measures of flowers included: labellum length (LL), labellum width (LW), the distance between stigma and labellum bottom (SL), and the distance between stamen and labellum bottom (AL) (Li et al. 2006). SL and AL were measured after longitudinal incision of the labellum (Li et al. 2006). The main measurement indices of insects were body length (BL) and thorax height (TH), which were directly measured by digital caliper (Li et al. 2006).

### 2.5 Effect of Pollination Modes on Fruiting Rates

Forty plants were randomly selected and bagged with nylon bags before flowering. At the beginning of flowering, the following five treatments were carried out, with eight plants being randomly selected for each treatment: (1) Natural control (NC): no emasculation, no bagging, free pollination; (2) Artificial self‐pollination (SF): emasculation, bagging, artificial self‐pollination with pollens from the same flower; (3) Artificial xenogamy (XN): emasculation, bagging, artificial cross‐pollination with pollens from flowers on different seedlings; (4) Natural self‐pollination (NS): bagging, no emasculation; (5) Observation on apomixis (AP): emasculation and bagging (Chen et al. 2012; Archer et al. 2021; Moreira et al. 2022). During pollination, pollens were deposited in the stigma with forceps, and the seed setting rate of each treatment was detected 1 month after pollination.

## 3 Results

### 3.1 Flowering Phenology and Flower Morphology

*C. shanxiense* flower began to bloom in late May. Each plant usually had 1 to 2 flowers. The flowering period of individual plants lasted for 12–16 days. The full flowering period (~60%) was from early June to late June, lasting for about 24 days. Pollen was mucilaginous and immobile for the first 4 days after anthesis. After 4 days, pollen was liquid and mobile. If the stigma was located below the pollen mass, the liquid pollen flowed under the action of gravity and touched the stigma (Figure 1b–d). In contrast, if the stigma was located above the pollen mass, the pollen was not able to touch the stigma (Figure 1e).

### 3.2 Observation of Pollination Process

Insects visited *C. shanxiense* flowers on sunny days, and the activity of visiting flowers mainly took place at 10:00–16:30 every day in 2020 and 2021. During the whole field pollination observation period, only 19 species of insects visited the flowers, belonging to Hymenoptera, Diptera, Lepidoptera, and Orthoptera, and most of them settled on the flowers rather than entered the labellum. However, only *Lasioglossum zonulum* entered the labellum of *C. shanxiense* on the third day (Figure 1f). The position of *L. zonulum* landing on the flower was not fixed. It landed on the petal three times, traveling along the petal to the outlet of the labellum, lingering around the pollens, then landed on the other petal and repeated. It landed on the labellum twice and then crawled inside, but then crawled out of the entrance and flew away. Only once did it enter the labellum, stay in the pollination channel for about 8 s, crawl along the bottom of the labellum, pass the position of the stigma and pollen mass, and finally climb out from the exit, with the pollen of *C. shanxiense* on its back (Figure 1g–i).

### 3.3 The Morphology of *C. shanxiense* and Pollinators

The functional morphology of *C. shanxiense* is shown in Table 1. The results showed that the distance between the stigma and the posterior wall of the labellum was 1.20 mm, and the distance between the pollen and the edge of the posterior wall of the labellum was 1.13 mm, both lower than the thorax height (2.24 mm) of *L. zonulum* . This suggested that as *L. zonulum* passed through the pollen, its back was very likely to touch the sticky pollen, and when it passed through the stigma again with the pollen on its back, the pollen was most likely to touch the stigma. Therefore, the floral functional structure measurements of *C. shanxiense* showed that *L. zonulum* may be its effective pollinator, and the observation also indicated that only *L. zonulum* could successfully carry away the pollen of *C. shanxiense*.

**TABLE 1:** Floral functional morphology of *C. shanxiense* and body size of *L. zonulum*.

*C. shanxiense*	mm	*L. zonulum*	mm
LL	7.47 ± 0.63	BL	7.27 ± 0.43
LW	6.75 ± 0.52	TH	2.24 ± 0.36
SL	1.20 ± 0.21		
AL	1.13 ± 0.12		

*Note:* The values shown are mean ± standard deviation.

Abbreviations: AL, the distance between stamen and labellum bottom; BL, body length; LL, labellum length; LW, labellum width; SL, the distance between stigma and labellum bottom; TH, thorax height.

### 3.4 Effect of Pollination Modes on Fruiting Rates

As shown in Table 2, the fruiting rate of *C. shanxiense* was 70%, that of artificial self‐pollination and cross‐pollination was up to 76.9% and 90%, respectively. The fruiting rate of natural self‐pollination of *C. shanxiense* was 66.7%, and the apomixis was 0.

**TABLE 2:** Effect of pollination mode on fruiting rates of *C. shanxiense*.

	Number of flowers	Number of fruits	Seed‐setting rate
NC	10	7	70.00%
SF	13	10	76.90%
XN	10	9	90%
NS	12	8	66.70%
AP	11	0	0

Abbreviations: AP, observation on apomixis; NC, natural control; NS, natural self‐pollination; SF, artificial self‐pollination; XN, artificial xenogamy.

## 4 Discussion

There are abundant and diverse forms of pollen (mass) in *Cypripedium* species. Some of which are plate‐like, such as *C. japonicum* , *C. acaule* , and *C. bardolphianum*, etc., and usually pollinators can take away the whole pollen mass (Chen et al. 2013). The pollen of some *Cypripedium* plants is very sticky, such as *C. calceolus* (Tremblay 1994), *C. macranthos* var. *rebunense* (Tremblay 1994; Sugiura et al. 2001, 2002), *C. guttatum* (Bänziger et al. 2005) and *C. shanxiense* investigated in this study, where only a portion of the pollen can be scraped away with each pollinator's visit, but not the entire pollen mass.

So far, the pollinator species and/or the reproductive characteristics have been investigated in 26 species of *Cypripedium*. Among these, 23 species have an unpaid pollination mode, while *C. subtropicum* has a paid pollination model (Jiang et al. 2021), and *C. diskinsonianum* and *C. passerinum* are self‐pollinated (Catling 1990). According to the phylogenetic relationship, *Cypripedium* evolved from those large, multi‐flowered, multi‐leaved, and high‐stem to less‐flowered, less‐leaved, and low‐stem (Cox et al. 1997; Holsinger 1997; Eccarius 2009). Sun (2005) superimposed pollinator groups in *Cypripedium* phylogenetic trees and suggested that the automatic self‐crossing species of *Cypripedium* was an ancestral group, which was a non‐monophyletic branch located at the top of the phylogenetic trees. Most of them were tall, leafless, and flower‐rich; species located in the middle branches, including Sect. Obtusipetala, Sect. Cypripedium, Sect. Arietinum, Sect. Acaulia, Sect. Flabellinervia, and Sect. Bifolia, are pollinated by bees or bumblebees. Species at the base of the phylogenetic trees, such as Sect. Sinppedilum and Sect. Trigonopedia, are pollinated by flies and are characterized by fewer flowers, fewer leaves, and lower growth. Based on the entire arytenoid system tree, the evolutionary system of *Cypripedium* evolved from self‐pollination to pollination by bees and then to pollination by flies.

Keddy et al. (1983) found tha*t C. passerinum* is capable of self‐pollination, marking the first instance of self‐pollination discovered in *Cypripedium*. This study found that *C. shanxiense* is also a self‐compatible species, capable of producing seeds without the need for insect pollination, and is not apomictic. Its pollen became liquid after 4 days of blooming. Under the action of gravity, the pollen flows out to the stigma, and there is an autonomous self‐pollination mechanism, which is similar to *Paphiopedilum parishii* anthers stepping onto the stigma for self‐fertilization (Chen et al. 2012; Chen and Liu 2014). Of course, this phenomenon should also fall under the category of delayed self‐pollination and prioritizing opportunities to ensure outcrossing, while serving as a means to circumvent the lack of pollinators (Archer et al. 2021). However, if the stigma is positioned higher than the pollen mass, self‐pollination cannot be accomplished. Therefore, the relative position of the stigma to the pollen mass becomes crucial for the success of self‐pollination, but the determinants of this positional relationship are not clear. In addition, it was observed that *L. zonulum* visited the flowers and took away the pollen from younger flowers (pollen from 4 days ago). Moreover, *L. zonulum* had a good size match with *C. shanxiense*; therefore, it was possible to deposit the pollen on the stigma to realize effective pollination. However, the visitation frequency was very low, despite *L. zonulum* being able to access the flowers freely through the labellum entrance and exit. Combined with the fact that the rate of natural heterogametic pollination in this study was 0%, it can be hypothesized that insect pollination is not the main mode of pollination in *C. shanxiense*.

Studies have shown that flower traits can be used to predict the species and behavior of pollinators according to the theory of pollination syndrome (Faegri and Pijl 1979). Namely, the closely related plant species with similar morphologic characteristics tend to share the same pollinators. For example, based on the fact that *Paphiopedilum villosum* and *P. parishii* are pollinated by syrphid flies and *P. dianthum* is morphologically similar to them, it was accurately predicted that *P. dianthum* also uses syrphid flies for pollination, being confirmed by experimental observations (Shi et al. 2007). Another example is the two related species of Subsect. Macrantha, *C. smithii* and *C. tibeticum*, both of which are pollinated by Bombus queens (Li and Luo 2009). However, the current study does not fit well with the above theory. In fact, *C. parviflorum* (Case and Bradford 2009), *C. candidum* (Bender 1985), *C. henryi* (Li et al. 2008) and *C. calceolus* (Braunschmid et al. 2017) in the same group as *C. shanxiense* are all pollinated by bees, unable to self‐pollinate. According to the theory of pollination syndrome, *C. shanxiense* is expected also to be pollinated only by bees. However, our study showed that insects were not needed if *C. shanxiense* was self‐pollinated, which did not exclude the role of insects. The results of this study challenged the previous misconception that *C. shanxiense* relies only on insect pollination and provided important information for designing its future protection strategy.

This is despite the fact that outcrossing is thought to be the main driving force behind pollination diversity in the reproductive system, leading angiosperms to develop a number of mechanisms to avoid self‐crossing and promote outcrossing (Holsinger 1997; Zhang et al. 2024; Ricci et al. 2024). However, self‐pollination is also believed to have many benefits, such as expanding new habitats, avoiding unreliable pollinators, maintaining local adaptability in plant populations, and automatically passing on genetic genes to future generations (Kalisz et al. 1999; Liu et al. 2005; Chen et al. 2012). Based on the results of this study, it is clear that *C. shanxiense* has mechanisms capable of delayed self‐pollination as a strategy for its conservation of population continuity in situations of low population density or in the absence or scarcity of pollinators.

However, this study also has certain limitations. For example, our observations were primarily focused on a single location and a single growing season, which may not fully represent the pollination ecology of *C. shanxiense* in different environments or across different years. The frequency of recording pollinator visitation behavior was also relatively limited, potentially underestimating the role of some pollinators. Future research could expand the survey scope to cover multiple geographical populations and multiple growing seasons to more comprehensively assess pollination dynamics. Furthermore, more precise methods (such as pollen marking, video surveillance, etc.) could be employed to quantify the contributions of different pollinators and to further investigate the efficiency of self‐pollination and its impact on population genetic structure. This information will help to formulate more accurate conservation and management measures for *C. shanxiense*.

## 5 Conclusion

In this study, we investigated the pollination biology of *C. shanxiense* for the first time. *L. zonulum* was able to visit flowers and obtain pollen, and its size characteristics in terms of pollination function largely corresponded to the flower structure of *C. shanxiense*. However, the frequency of flower visits was extremely low, so insect pollination was not the main pollination method of *C. shanxiense*. *C. shanxiense* was highly self‐compatible, with pollen turning from mucilaginous to liquid after 4 days of flowering, flowing by gravity into the stigma and self‐pollinating to produce seeds. Delayed self‐pollination allows *C. shanxiense* to adapt to sheltered ex situ environments where pollinators are not essential, but their presence forms the basis for heterogamous pollination and its beneficial effects. Since the determinants of the relative position of the stigma and pollen mass are unknown, an in‐depth study of this is necessary. Future research needs to expand survey coverage and duration, adopt more precise methods to quantify pollinator contributions, and delve into the efficiency of self‐pollination and its effects on population genetics. This will provide a more solid foundation for managing the conservation of *C. shanxiense*.

## Conflicts of Interest

The authors declare no conflicts of interest.

## Supporting information


Data S1.

## Data Availability

Data used in the study are available from the Supporting Information.
